# Preoperative albumin-to-globulin ratio and prognostic nutritional index predict the prognosis of colorectal cancer: a retrospective study

**DOI:** 10.1038/s41598-023-43391-5

**Published:** 2023-10-12

**Authors:** JunHu Li, Na Zhu, Cheng Wang, LiuPing You, WenLong Guo, ZhiHan Yuan, Shuai Qi, HanZheng Zhao, JiaYong Yu, YueNan Huang

**Affiliations:** 1 Department of General Surgery, The Second Affiliated Hospital of Harbin Medical University, Harbin, China; 2 Department of Phase I Clinical Research, Tianjin Medical University Cancer Institute and Hospital, Tianjin, China

**Keywords:** Cancer, Oncology

## Abstract

The immunonutritional status has important effects on outcomes for cancer patients. Albumin-to-globulin ratio (AGR) and the prognostic nutrition index (PNI) are often used to assess the immunonutritional status of cancer patients. However, the clinical significance of these factors in colorectal cancer (CRC) remains unclear. We aimed to evaluate the clinical significance of the AGR and PNI in CRC. We reviewed the clinical data of 511 patients with CRC in two hospitals. Data from one institution was used as the training cohort. The optimal cutoff values for AGR and PNI in the training cohort were 1.4 and 48.65, respectively. Patients in both the low AGR and low PNI groups had poor overall survival (OS) and progression-free survival (PFS), while those in the low AGR-low PNI group had the lowest OS and PFS. Multivariate analysis revealed that preoperative AGR, preoperative PNI, gross type, and TNM stage were independent prognostic factors influencing OS in patients with CRC. Preoperative AGR, preoperative PNI, and TNM stage were independently associated with PFS in patients with CRC. According to the results of multivariate analysis in the training cohort, we developed the nomograms for OS and PFS and performed internal and external validation, which showed good prediction ability of the nomograms. In conclusion, preoperative AGR and PNI can be used as effective indicators to predict survival for patients with CRC. AGR and PNI may help develop effective adjuvant-therapy schedules.

## Introduction

Colorectal cancer (CRC) is the third most common malignancy and its mortality rate is the second highest among cancer-related mortality in the world^1^. In China, CRC is the third most common tumor, with the fifth highest mortality rate^2^. And its incidence continues to increase^2^. Despite significant improvements in the treatment and management, the prognosis remains poor for patients with CRC. Relevant data showed that the 5-year survival rate of CRC patients was about 62%, while the 5-year survival rate of patients with metastatic CRC was only 11%^3^. Colorectal cancer has become the serious global public health problem, and identifying effective prognostic biomarkers could significantly improve the prognosis of patients with CRC and make more personalized and targeted treatment strategies.

TNM stage (T stage, local tumor spread; N stage, lymph node spread; M stage, metastasis), carcinoembryonic antigen (CEA) and carbohydrate antigen 19-9 (CA19-9) play an important role in evaluating the prognosis of CRC and guiding treatment options^4,5^. However, the prognosis of patients cannot be fully explained by currently established prognostic factors, including TNM stage and CEA. This means that more prognostic factors should be taken into account, such as nutritional status and immune status.

Many studies had shown that nutritional status and systemic inflammatory response were important factors in tumor development and prognosis^6–8^. Nutrition is associated with many aspects of tumor development and plays an important role in throughout the treatment process. Malnutrition is common in patients with CRC, which can suppress immune function and reduce resistance to disease^9,10^.

Albumin (ALB) and globulins (GLB), the main components of serum proteins, had been proved to involve in the development of systemic inflammation and could be widely used to assess nutritional status and disease severity in cancer patients^11,12^. The low level of ALB or high level of GLB are often associated with high mortality and recurrence rates in many types of cancer^13,14^. Therefore, the cumulative effect of ALB and GLB can provide effective prognostic value for cancer patients.

Decreased nutritional status in cancer patients is also associated with systemic inflammatory responses^15^. Immuno-inflammatory biomarkers (IIBs) can reflect the balance between the status of inflammation and immunity in the host, and predict the prognosis of patients with CRC, including neutrophil-to-lymphocyte ratio (NLR), platelet-to-lymphocyte ratio (PLR), and pan-immune inflammation value (PIV)^16–18^. Lymphocytes are an important component of the immune system and are important effector cells for anti-tumor immunity^19^. The low absolute counts of lymphocyte had been proved to be associated with poor prognosis of CRC^19^.

However, serum ALB, GLB level, and lymphocyte counts are susceptible to confounding factors, which limits their accuracy in reflecting the nutritional and inflammatory status of the patient. To overcome this deficiency and better reflect the systemic immunonutritional status, we introduce albumin-to-globulin ratio (AGR) and prognostic nutritional index (PNI) in this study.

Albumin-to-globulin ratio (AGR) is the combination of nutritional and inflammatory indicator, and its high expression level is closely associated with longer survival time in cancer patients^20^. AGR had been found to be an effective prognostic factor for many types of cancer, including colorectal, gastric, head and neck, and breast^21–24^. Prognostic nutritional index (PNI) can reflect the chronic inflammation, immune status, and nutritional status of the patients with cancer, which is often used to predict the risk of complications after gastrointestinal surgery^25,26^. Many studies had shown that PNI was associated with survival of various types of cancer, such as breast, lung, colorectal, gastric cancer^25–28^.

However, most studies focused on the prognostic value of either AGR or PNI for various types of cancer, but a single marker might not accurately predict the prognosis of patients with CRC. Currently, the prognostic value of AGR combined with PNI in patients of CRC has not been investigated. This study aimed to evaluate the association between preoperative AGR and PNI with the clinicopathological features of CRC, and to investigate the prognostic value of these two indicators in patients with CRC.

## Patients and methods

### Patients

This study retrospectively selected 396 patients with primary CRC who underwent radical surgery at the Second Affiliated Hospital of Harbin Medical University and 115 patients with primary CRC who underwent radical surgery at the First Affiliated Hospital of Harbin Medical University between December 2016 and June 2018. The inclusion criteria were: (1) patients had primary CRC confirmed by postoperative histopathology; (2) patients received radical surgery; (3) the age of the patient was > 18 years old; (4) patients had complete and reliable clinical data, and could complete follow-up. The exclusion criteria were: (1) patients had non-primary CRC; (2) patients had history of other malignancies or were concomitant with other primary cancers; (3) patients were accompanied by unresectable distant metastases; (4) patients had hematologic diseases and autoimmune diseases; (5) patients had severe liver and kidney dysfunction, or other diseases that caused poor nutritional status; (6) patients had infection or other inflammatory diseases before surgery; (7) patients underwent radiotherapy or chemotherapy before surgery; (8) patients received parenteral nutrition support therapy within 2 weeks before surgery.

396 patients from the Second Affiliated Hospital of Harbin Medical University were used as the training cohort to explore prognostic factors and construct the nomograms. 115 patients from the First Affiliated Hospital of Harbin Medical University were used as the validation cohort for the verification of nomograms.

This study was conducted in compliance with the postulates of Declaration of Helsinki and approved by the Human Ethics Committee of the Second Affiliated Hospital of Harbin Medical University (YJSKY2022-184). The requirement for patient approval or informed consent was waived by the Human Ethics Committee of the Second Affiliated Hospital of Harbin Medical University, owing to the retrospective nature of the study and because the analysis used anonymous clinical data.

### Data collection

The following variables were collected: gender, age, CEA, CA19-9, tumor location, tumor size, tumor gross type, histological type, vascular invasion, nerve invasion, lymphatic infiltration, T stage, N stage, M stage, TNM stage, surgical approach, chemotherapy, operative time and bleeding volume. Tumor staging was performed according to the 7th edition of the Union for International Cancer Control-American Joint Committee on cancer classification for CRC.

Serum albumin (ALB), total protein, and blood counts, including lymphocytes, neutrophils, monocytes, and platelets, which were collected from the preoperative blood of patients. NLR, PLR, PIV, AGR and PNI were calculated in turn. NLR = neutrophil count (10^9^/L)/lymphocyte count (10^9^/L); PLR = platelet count (10^9^/L) / lymphocyte count (10^9^/L); PIV = [neutrophil count (10^9^/L) × platelet count (10^9^/L) × monocyte count (10^9^/L)]/lymphocyte count (10^9^/L); AGR = albumin/(total protein−albumin), PNI = albumin (g/L) + 5 × total lymphocyte count (10^9^/L).

### Follow‑up

All included patients underwent rigorous follow-up. Routine follow-up of patients was performed every 3 months during the first 2 years after the operation, every 6 months after 2 years, and every year after 5 years. Follow-up examinations include routine laboratory examinations, computed tomography (CT) or magnetic resonance imaging (MRI), and an annual colonoscopy. Positron emission tomography scan was used to better identify tumor recurrence or metastasis, when necessary. Survival information and outcomes were obtained from clinical records or household contact during follow-up. The end time of follow-up is until the patient dies or loses to follow-up, or by March 2023. Patients with a survival time of 0 months were excluded. The overall survival (OS) was defined as the time from the date of diagnosis to death or last follow-up. The progression-free survival (PFS) was defined as the time from the date of diagnosis to progression, relapse, death, or last follow-up, whichever came first.

### Statistical analysis

Statistical analysis was performed using R programming language (v 4.2.0). We performed statistical analysis based on data from 396 patients in the training cohort. The receiver-operating characteristic (ROC) curve analysis was performed to determine the optimal cutoff values for AGR and PNI. And the area under the ROC curve (AUC) was calculated to assess the predictive ability of AGR and PNI. The between-group difference analysis was performed based on the R package “compareGroups” (v 4.4.5). Continuous variables were analyzed using Student t-test or Mann–Whitney U test. Categorical variables were analyzed by the χ2 test or Fisher's exact test. Continuous variables were shown as medians or interquartile ranges. Categorical variables were expressed as absolute numbers or percentages. Survival outcomes were analyzed using the Kaplan–Meier method and compared using log-rank tests. The risk factors for OS and PFS were determined by Cox proportional hazards regression analysis, and expressed using hazard ration (HR) with a 95% confidence interval (CI).

Based on the results of Cox regression analysis, the R package "rms" (v 6.3-0) was used to build the nomogram models to predict the prognosis of CRC patients. Next, the nomograms underwent internally validation using the bootstrap method. The receiver operating characteristic (ROC) curve and calibration curve were used to evaluate the discrimination ability and prediction effect of nomograms. Moreover, we performed external validation by validating 115 patients in the validation cohort to evaluate the performance of the nomograms. Unless otherwise stated, all statistical tests were two-sided and p < 0.05 was considered statistically significant.

### Ethics approval and consent to participate

This study conformed to the assumptions of the Declaration of Helsinki and was approved by the Human Ethics Committee of the Second Affiliated Hospital of Harbin Medical University (YJSKY2022-184). The requirement for patient approval or informed consent was waived by the Human Ethics Committee of the Second Affiliated Hospital of Harbin Medical University, owing to the retrospective nature of the study and because the analysis used anonymous clinical data.

## Results

### Clinicopathologic characteristics of patients

Table 1 summarizes the clinicopathological characteristics of all patients. Between December 2016 and June 2018, a total of 511 patients were recruited from the Second Affiliated Hospital of Harbin Medical University (training cohort, n = 396) and the First Affiliated Hospital of Harbin Medical University (validation cohort, n = 115). The demographic and clinical factors were largely consistent between the training and validation cohorts. The median follow-up for these patients was 64.0 months (range 2.0–134.0 months). Patients included more male than female (62.6% vs 37.4%), and the median age was 61 years (range 25–88 years). More than half of patients had a primary tumor located in the rectum (50.1%). The majority of patients (79.8%) had moderately differentiated histological types, followed by the poorly differentiated (15.1%) and the highly differentiated (5.1%). And the numbers of patients with ulcerative tumor and protrude tumor were 373 (73.0%) and 138 (27.0%), respectively. Additionally, patients with TNM stage I, II, III, and IV tumors accounted for 16.0%, 44.2%, 36.7% and 3.1% of all cases, respectively. There were 434 patients whose pathological results included vascular invasion, nerve invasion and lymphatic infiltration. Among them, positive patients were 160 (31.3%), 307 (60.0%) and 119 (23.3%), respectively. Open surgery was performed in 382 patients (74.8%) and laparoscopic surgery was performed in 129 patients (25.2%). A total of 61.3% of patients received adjuvant chemotherapy.

**Table 1:** Clinicopathologic characteristic of patients with CRC.

Characteristics	Total (n = 511)	Training cohort (n = 396)	Validation cohort (n = 115)
	No. of patients (%)	No. of patients (%)	No. of patients (%)
Gender			
Male	320 (62.6%)	242 (61.1%)	78 (67.8%)
Female	191 (37.4%)	154 (38.9%)	37 (32.2%)
Age			
< 61 years	242 (47.4%)	192 (48.5%)	50 (43.5%)
≥ 61 years	269 (52.6%)	204 (51.5%)	65 (56.5%)
Tumor location			
Colon	255 (49.9%)	193 (48.7%)	62 (53.9%)
Rectum	256 (50.1%)	203 (51.3%)	53 (46.1%)
Histological type			
High	26 (5.1%)	21 (5.3%)	5 (4.4%)
Medium	408 (79.8%)	324 (81.8%)	84 (73.0%)
Low	77 (15.1%)	51 (12.9%)	26 (22.6%)
Gross type			
Ulcerative	373 (73.0%)	279 (70.5%)	94 (81.7%)
Protrude	138 (27.0%)	117 (29.5%)	21 (18.3%)
TNM stage			
I	82 (16.0%)	80 (20.2%)	2 (1.7%)
II	226 (44.2%)	182 (46.0%)	44 (38.3%)
III	187 (36.7%)	129 (32.5%)	58 (50.4%)
IV	16 (3.1%)	5 (1.3%)	11 (9.6%)
Vascular invasion			
+	160 (31.3%)	111 (28.0%)	49 (42.6%)
−	274 (53.6%)	222 (56.1%)	52 (45.2%)
NA	77 (15.1%)	63 (15.9%)	14 (12.2%)
Nerve invasion			
+	307 (60.0%)	228 (57.6%)	79 (68.7%)
−	127 (24.9%)	105 (26.5%)	22 (19.1%)
NA	77 (15.1%)	63 (15.9%)	14 (12.2%)
Lymphatic infiltration			
+	119 (23.3%)	82 (20.7%)	37 (32.1%)
−	315 (61.6%)	251 (63.4%)	64 (55.7%)
NA	77 (15.1%)	63 (15.9%)	14 (12.2%)
Surgical approach			
Open	382 (74.8%)	300 (75.8%)	82 (71.3%)
Laparoscopy	129 (25.2%)	96 (24.2%)	33 (28.7%)
Chemotherapy			
Yes	313 (61.3%)	239 (60.4%)	74 (64.3%)
No	198 (38.7%)	157 (39.6%)	41 (35.7%)

NA, not available.

### Determine the optimal cutoff values of AGR and PNI in the training cohort

**Figure 1:**
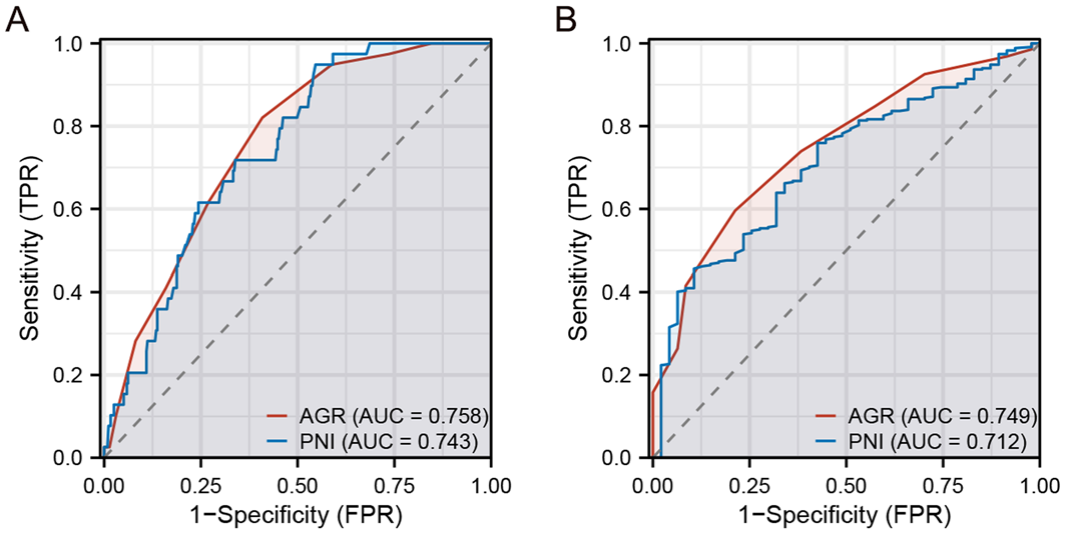
(**A**) ROC curves for AGR and PNI in overall survival from the training cohort (the cutoff value: 1.4 and 48.65; AUC: 0.758 and 0.743; P < 0.001). (**B**) ROC curves for AGR and PNI in progression-free survival from the training cohort (the cutoff value: 1.3 and 48.65; AUC: 0.749 and 0.712; P < 0.001).

In the training cohort, the median preoperative AGR and PNI were 1.5 and 51.75, respectively. With ROC analysis of overall survival (OS) of the training cohort, the optimal cut-off values for AGR and PNI were 1.4 and 48.65, respectively (Fig. 1A). ROC analysis for progression-free survival (PFS) showed optimal cutoff values of 1.3 and 48.65 for AGR and PNI, respectively (Fig. 1B). The optimal cut-off value obtained by ROC analysis of OS had great statistical significance and was convenient for clinical application. In this study, we chose the optimal cut-off values AGR (1.4) and PNI (48.65) obtained by ROC analysis of OS for analysis.

### Association of clinical characteristics with preoperative AGR and PNI in the training cohort

As shown in Table 2, the differences between preoperative AGR and PNI in age (p < 0.001, p = 0.008), PLR (p < 0.001, p < 0.001), NLR (p = 0.011, p < 0.001), PIV (p < 0.001, p < 0.001), tumor location (p < 0.001, p < 0.001), tumor size (p < 0.001, p < 0.001) were statistically significant. In addition, preoperative AGR was significantly associated with TNM stage (p = 0.013), while preoperative PNI was significantly associated with T stage (p = 0.027). However, there were no significant differences between preoperative AGR and PNI in terms of gender, CEA, CA19-9, histological type, gross type, pathological N stage, M stage, vascular invasion, nerve invasion, lymphatic infiltration, surgical approach, operative time and bleeding volume. We found that preoperative low AGR and PNI were associated with aggressive clinicopathological features in CRC patients, such as high age, high PLR, high NLR, high PIV, high TNM stage, and large tumor size.

**Table 2:** Clinicopathological characteristic of patients with CRC stratified by AGR and PNI cutoffs in the training cohort.

Characteristics	All n = 396	AGR		p-value	PNI		p-value
		Low n = 120	High n = 276		Low n = 111	High n = 285	
Gender, n (%)				0.331			0.379
Male		69 (17.4%)	173 (43.7%)		64 (16.2%)	178 (44.9%)	
Female		51 (12.9%)	103 (26.0%)		47 (11.9%)	107 (27.0%)	
Age, median (IQR)		63.50 (55.75, 70.00)	60.00 (53.00, 65.25)	< 0.001	63.00 (54.00, 69.00)	60.00 (53.00, 66.00)	0.008
CEA, median (IQR)		3.16 (1.74, 8.00)	3.20 (2.00, 5.83)	0.762	2.96 (1.76, 7.07)	3.21 (2.00, 5.94)	0.983
CA19-9, median (IQR)		7.46 (3.94, 20.00)	7.77 (4.04, 15.22)	0.847	6.22 (3.46, 14.86)	8.67 (4.12, 18.37)	0.080
PLR, median (IQR)		154.20 (112.21, 206.83)	126.33 (100.47, 157.77)	< 0.001	175.79 (141.21, 239.26)	119.46 (95.96, 151.68)	< 0.001
NLR, median (IQR)		2.16 (1.63, 2.93)	1.90 (1.45, 2.58)	0.011	2.51 (1.86, 3.68)	1.82 (1.40, 2.42)	< 0.001
PIV, median (IQR)		221.67 (132.29, 406.59)	137.39 (86.80, 249.65)	< 0.001	221.74 (114.52, 460.95)	145.12 (97.27, 251.23)	< 0.001
Tumor location, n (%)				< 0.001			< 0.001
Colon		77 (19.4%)	116 (29.3%)		76 (19.2%)	117 (29.5%)	
Rectum		43 (10.9%)	160 (40.4%)		35 (8.8%)	168 (42.4%)	
Tumor size, median (IQR)		5.00 (3.50, 6.25)	4.00 (3.00, 5.00)	< 0.001	5.00 (3.60, 6.45)	4.00 (3.00, 5.00)	< 0.001
Histological type, n (%)				0.784			0.440
High		5 (1.3%)	16 (4.0%)		5 (1.3%)	16 (4.0%)	
Medium		100 (25.3%)	224 (56.6%)		88 (22.2%)	236 (59.6%)	
Low		15 (3.8%)	36 (9.1%)		18 (4.5%)	33 (8.3%)	
Gross type, n (%)				0.727			0.660
Ulcerative		86 (21.7%)	193 (48.7%)		80 (20.2%)	199 (50.3%)	
Protrude		34 (8.6%)	83 (21.0%)		31 (7.8%)	86 (21.7%)	
T stage, n (%)				0.056			0.027
T1		5 (1.3%)	17 (4.3%)		5 (1.3%)	17 (4.3%)	
T2		15 (3.8%)	58 (14.6%)		16 (4.0%)	57 (14.4%)	
T3		46 (11.6%)	74 (18.7%)		46 (11.6%)	74 (18.7%)	
T4		54 (13.6%)	127 (32.1%)		44 (11.1%)	137 (34.6%)	
N stage, n (%)				0.945			0.093
N0		79 (19.9%)	185 (46.7%)		66 (16.7%)	198 (50.0%)	
N1		31 (7.8%)	67 (16.9%)		31 (7.8%)	67 (16.9%)	
N2		10 (2.5%)	24 (6.1%)		14 (3.5%)	20 (5.1%)	
M stage, n (%)				0.052			0.366
M0		116 (29.3%)	275 (69.4%)		111 (28%)	280 (70.7%)	
M1		4 (1.0%)	1 (0.3%)		0 (0.0%)	5 (1.3%)	
TNM stage, n (%)				0.013			0.076
I		16 (4.0%)	64 (16.2%)		17 (4.3%)	63 (15.9%)	
II		61 (15.4%)	121 (30.6%)		49 (12.4%)	133 (33.6%)	
III		39 (9.8%)	90 (22.7%)		45 (11.4%)	84 (21.2%)	
IV		4 (1.0%)	1 (0.3%)		0 (0.0%)	5 (1.3%)	
Vascular invasion^a^, n (%)				0.929			0.715
+		32 (9.6%)	78 (23.4%)		33 (9.9%)	77 (23.1%)	
−		67 (20.1%)	156 (46.8%)		60 (18.0%)	163 (48.9%)	
Nerve invasion^a^, n (%)				0.754			0.256
+		69 (20.7%)	159 (47.7%)		68 (20.4%)	160 (48.0%)	
−		30 (9.0%)	75 (22.5%)		25 (7.5%)	80 (24.0%)	
Lymphatic infiltration^a^, n (%)				0.466			0.590
+		27 (8.1%)	55 (16.5%)		21 (6.3%)	61 (18.3%)	
−		72 (21.6%)	179 (53.8%)		72 (21.6%)	179 (53.8%)	
Surgical approach, n (%)				0.444			0.448
Open		90 (22.7%)	210 (53.0%)		87 (22.0%)	213 (53.8%)	
Laparoscopy		30 (7.6%)	66 (16.7%)		24 (6.1%)	72 (18.2%)	
Operative time, median (IQR)		150 (125, 175)	150 (125, 180)	0.997	150 (130, 180)	150 (120, 175)	0.724
Bleeding volume, median (IQR)		150 (80, 200)	100 (50, 200)	0.100	100 (85, 200)	100 (50, 200)	0.217

^a^Missing in 63 patients.

### Association of AGR and PNI with survival in the training cohort

**Figure 2:**
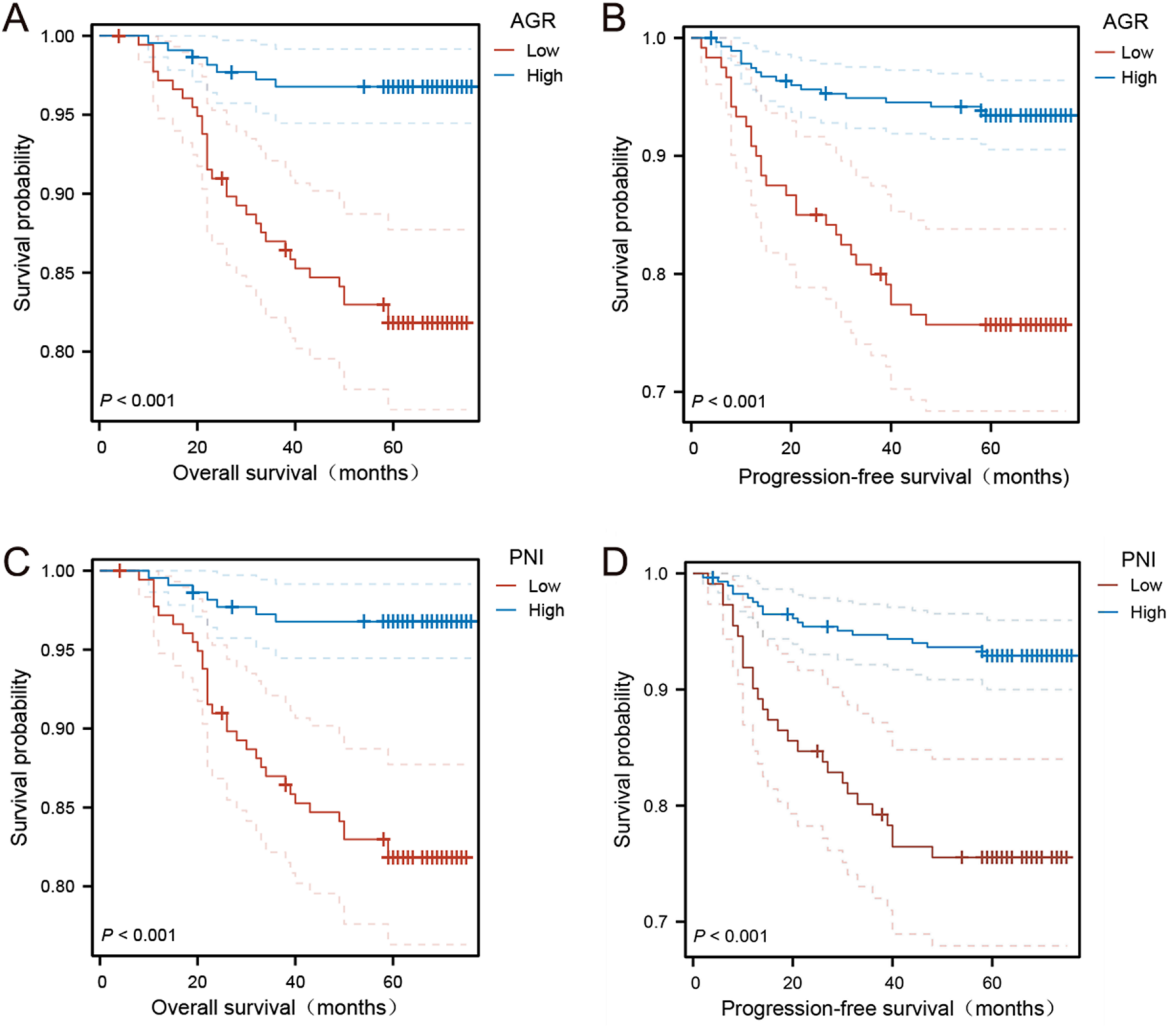
(**A**) Kaplan–Meier survival curves of overall survival according to AGR in the training cohort (P < 0.001). (**B**) Kaplan–Meier survival curves of progression-free survival according to AGR in the training cohort (P < 0.001). (**C**) Kaplan–Meier survival curves of overall survival according to PNI in the training cohort (P < 0.001). (D) Kaplan–Meier survival curves of progression-free survival according to PNI in the training cohort (P < 0.001).

In the training cohort, 357 patients (90.2%) still alive at the last follow-up and 47 patients (11.9%) experienced recurrences. The median OS and PFS for all patients in the training cohort were 67.0 and 67.0 months, respectively. We further found that the median OS and PFS of patients in the high AGR group were significantly higher than those in the low AGR group. High preoperative AGR was associated with significant improvements in OS and PFS, and this difference in survival was statistically significant (Fig. 2A and B, p < 0.001). Similarly, patients with a higher PNI were associated with the significantly improved OS and PFS. And this difference in survival was also statistically significant (Fig. 2C and D, p < 0.001).

**Figure 3:**
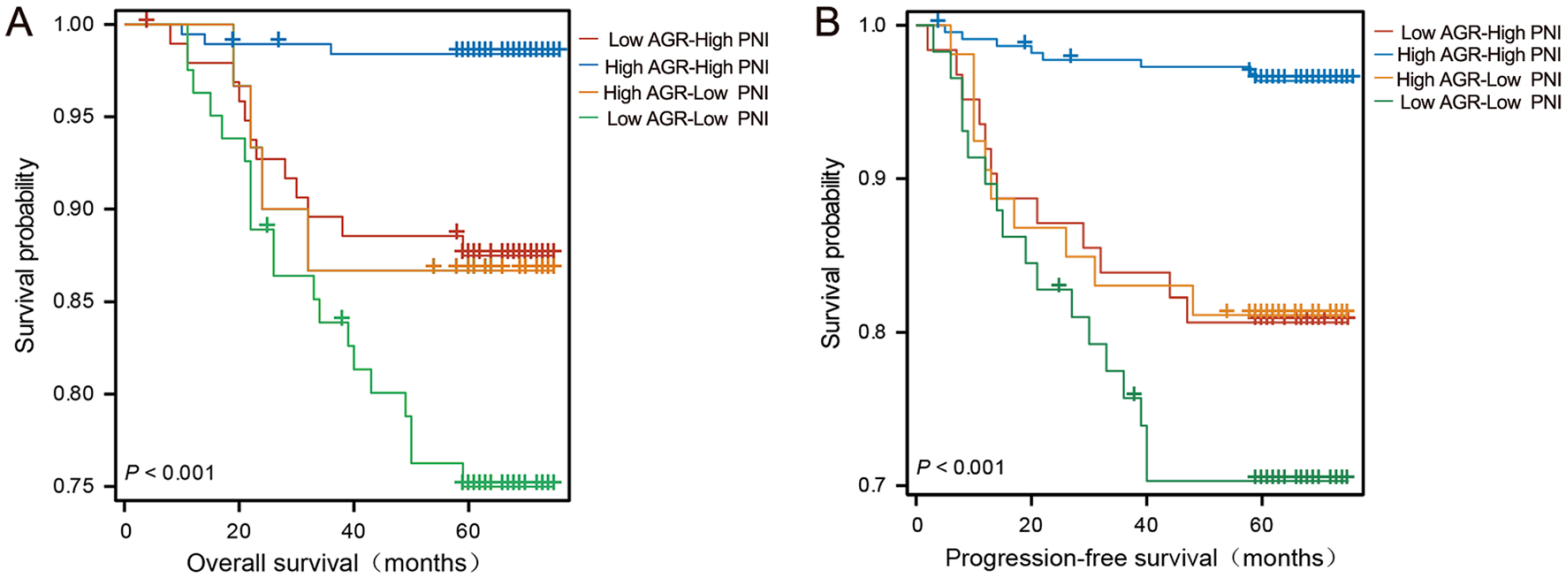
(**A**) Kaplan–Meier survival curves of overall survival according to both AGR and PNI in the training cohort (P < 0.001). (**B**) Kaplan–Meier survival curves of progression-free survival according to both AGR and PNI in the training cohort (P < 0.001).

In order to further investigate the predictive value of AGR combined with PNI in CRC, we performed joint analysis of AGR and PNI, which was shown in Fig. 3. The median OS and PFS of patients in the high AGR-high PNI group were 67.5 months and 67.0 months, respectively, which were higher than those in the high AGR-low PNI group, low AGR-high PNI group, and low AGR-low PNI group. Patients in the high AGR-high PNI group had the highest OS and PFS rates than those in other groups, and this difference in survival was statistically significant (Fig. 3A and B, p < 0.001).

### Prognostic factors for OS and PFS in the training cohort

Table 3 summarized the effects of clinicopathological variables on OS in CRC patients in the training cohort. The univariate analysis demonstrated that preoperative AGR, preoperative PNI, histological type, gross type, and TNM stage were significantly associated with OS in CRC patients (p < 0.05). Cox regression analysis demonstrated that preoperative AGR, preoperative PNI, gross type and TNM stage were independently associated with OS in CRC patients. Patients with preoperative low AGR had a lower incidence of OS than patients with preoperative high AGR (HR: 3.809, 95% CI: 1.595–9.094, p = 0.003). Similarly, patients with low preoperative PNI had lower overall survival (HR: 2.782, 95% CI: 1.356 to 5.709, p = 0.005). Meanwhile, we found that patients with ulcerative CRC also had lower OS than patients with protrude CRC (HR: 4.935, 95% CI: 1.162 to 20.962, p = 0.031). In addition, patients with higher TNM stage had lower overall survival. Patients with TNM-III (HR: 2.002, 95% CI: 1.012–3.961, p = 0.046) and TNM-IV (HR: 5.209, 95%CI: 1.075–25.252, p = 0.040) had worse prognosis compared with patients with TNM-II.

**Table 3:** Cox regression analysis of predictors for overall survival in the training cohort.

Characteristics	Univariate analysis			Multivariate analysis		
	HR	95% CI	p-value	HR	95% CI	p-value
Gender			0.695			
Male	Reference					
Female	0.878	0.456–1.689	0.697			
Age	1.028	0.996–1.061	0.084			
AGR			< 0.001			
High	Reference			Reference		
Low	6.038	2.665–13.682	< 0.001	3.809	1.595–9.094	0.003
PNI			< 0.001			
High	Reference			Reference		
Low	4.426	2.321–8.439	< 0.001	2.782	1.356–5.709	0.005
CA19-9	1.001	0.998–1.003	0.667			
NLR	1.091	0.966–1.233	0.162			
Tumor location			0.186			
Rectum	Reference					
Colon	1.531	0.809–2.899	0.190			
Tumor size	1.092	0.934–1.276	0.268			
Histological type			0.013			
High	0.643	0.387–15.672	0.996	0.562	0.254–12.267	0.997
Medium	Reference			Reference		
Low	2.315	1.128–4.751	0.022	1.700	0.819–3.530	0.154
Gross type			< 0.001			
Protrude	Reference			Reference		
Ulcerative	8.341	2.010–34.610	0.003	4.935	1.162–20.962	0.031
TNM stage			< 0.001			
I	0.168	0.022–1.285	0.086	0.330	0.042–2.572	0.290
II	Reference			Reference		
III	2.624	1.329–5.181	0.005	2.002	1.012–3.961	0.046
IV	6.646	1.500–29.451	0.013	5.209	1.075–25.252	0.040
Vascular invasion^a^			0.122			
−	Reference					
+	1.774	0.866–3.636	0.117			
Nerve invasion^a^			0.055			
+	Reference					
−	0.423	0.162–1.104	0.079			
Lymphatic infiltration^a^			0.266			
−	Reference					
+	1.558	0.729–3.328	0.252			
Operative time	1.003	0.996–1.009	0.405			
Chemotherapy			0.055			
No	Reference					
Yes	0.545	0.290–1.023	0.059			
Surgical approach			0.547			
Open	Reference					
Laparoscopy	0.792	0.364–1.722	0.556			

^a^Missing in 63 patients.

The effects of clinicopathological variables on PFS in patients with CRC of the training cohort were shown in Table 4. On univariate analysis, preoperative AGR, preoperative PNI, gross type, TNM stage, and nerve invasion were confirmed to be significantly associated with PFS in CRC patients (p < 0.05). Multivariate COX regression analysis showed that preoperative AGR, preoperative PNI, and TNM stages were independently associated with PFS in CRC patients. Patients with low preoperative AGR had lower PFS compared with patients with high preoperative AGR (HR: 2.584, 95% CI: 1.300–5.138, p = 0.007). Patients with low preoperative PNI had a lower PFS rate (HR: 2.332, 95% CI: 1.172 to 4.640, p = 0.016). Meanwhile, we found that patients with high TNM stage had lower progression-free survival. Patients with TNM-III (HR: 2.107, 95% CI: 1.058–4.196, p = 0.034) had worse prognosis than patients with TNM-II.

**Table 4:** Cox regression analysis of predictors for progression-free survival in the training cohort.

Characteristics	Univariate analysis			Multivariate analysis		
	HR	95% CI	p-value	HR	95% CI	p-value
Gender			0.937			
Female	Reference					
Male	1.024	0.569–1.844	0.937			
Age	1.014	0.986–1.043	0.327			
AGR			< 0.001			
High	Reference			Reference		
Low	4.092	2.272–7.370	< 0.001	2.584	1.300–5.138	0.007
PNI			< 0.001			
High	Reference			Reference		
Low	3.908	2.191–6.971	< 0.001	2.332	1.172–4.640	0.016
CEA	0.999	0.992–1.006	0.781			
NLR	1.083	0.964–1.216	0.178			
Tumor location			0.114			
Colon	Reference					
Rectum	0.628	0.351–1.125	0.118			
Tumor size	1.083	0.940–1.248	0.271			
Histological type			0.066			
High	0.422	0.058–3.082	0.395			
Medium	Reference					
Low	2.111	1.072–4.158	0.031			
Gross type			< 0.001			
Ulcerative	Reference			Reference		
Protrude	0.262	0.104–0.663	0.005	0.487	0.168–1.414	0.186
TNM stage			< 0.001			
I	0.254	0.059–1.100	0.067	0.372	0.046–2.999	0.353
II	Reference			Reference		
III	2.299	1.247–4.238	0.008	2.107	1.058–4.196	0.034
IV	5.379	1.242–23.288	0.024	2.967	0.365–24.093	0.309
Vascular invasion^a^			0.096			
−	Reference					
+	1.745	0.914–3.330	0.092			
Nerve invasion^a^			0.026			
+	Reference			Reference		
−	0.406	0.169–0.973	0.043	0.684	0.276–1.698	0.413
Lymphatic infiltration^a^			0.474			
+	Reference					
−	0.769	0.380–1.557	0.466			
Operative time	1.003	0.997–1.009	0.412			
Chemotherapy			0.276			
No	Reference					
Yes	0.728	0.411–1.291	0.278			
Surgical approach			0.624			
Open	Reference					
Laparoscopy	0.842	0.419–1.693	0.630			

^a^Missing in 63 patients.

### Construction and validation of nomograms for OS and PFS in CRC

**Figure 4:**
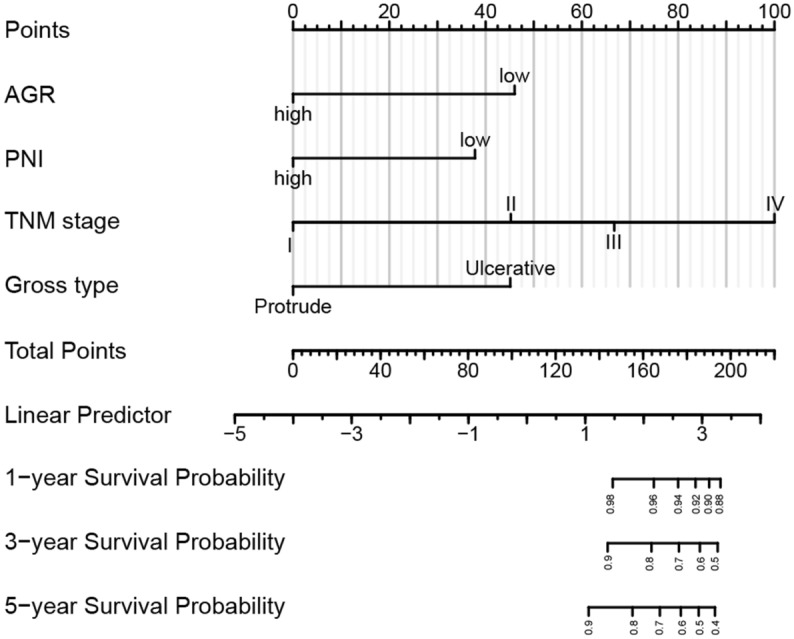
Nomogram model predicting overall survival from the training cohort. The nomogram is used by summing all points identified on the scale for each variable. The total points projected on the bottom scales indicate the probabilities of 1-, 3- and 5-year survival.

**Figure 5:**
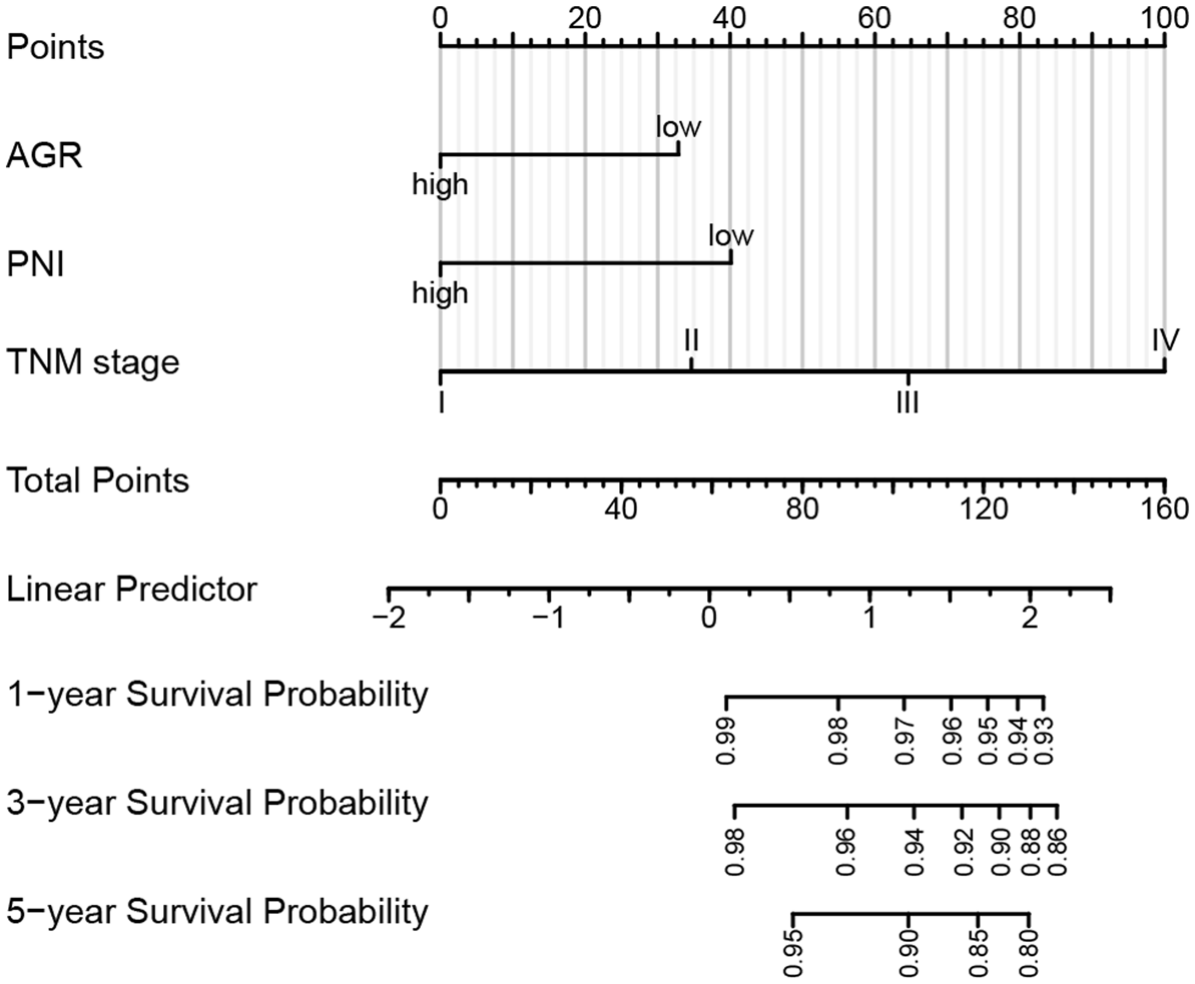
Nomogram model predicting progression-free survival from the training cohort. The nomogram is used by summing all points identified on the scale for each variable. The total points projected on the bottom scales indicate the probabilities of 1-, 3- and 5-year survival.

Based on the results of the above COX regression analysis in the training cohort, we constructed the nomogram models for OS and PFS in CRC patients to further analyze the influence of these factors on patient prognosis (Figs. 4 and 5). According to the nomogram models, we found that preoperative AGR and TNM stage conferred the greater effect on OS of patients with CRC (Fig. 4). Specifically, patients with CRC with high preoperative AGR and low TNM stage had higher rates of OS. Meanwhile, we found that preoperative PNI and TNM stage had the greater effect on PFS and the greater contribution to risk scores in CRC patients (Fig. 5). Patients with low preoperative PNI and high TNM stage had lower incidences of PFS.

**Figure 6:**
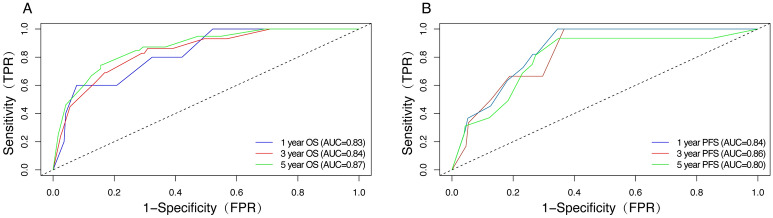
(**A**) ROC curves for 1-, 3-, and 5-year OS based on the nomogram for the training cohort. (**B**) ROC curves for 1-, 3-, and 5-year PFS based on the nomogram for the training cohort.

**Figure 7:**
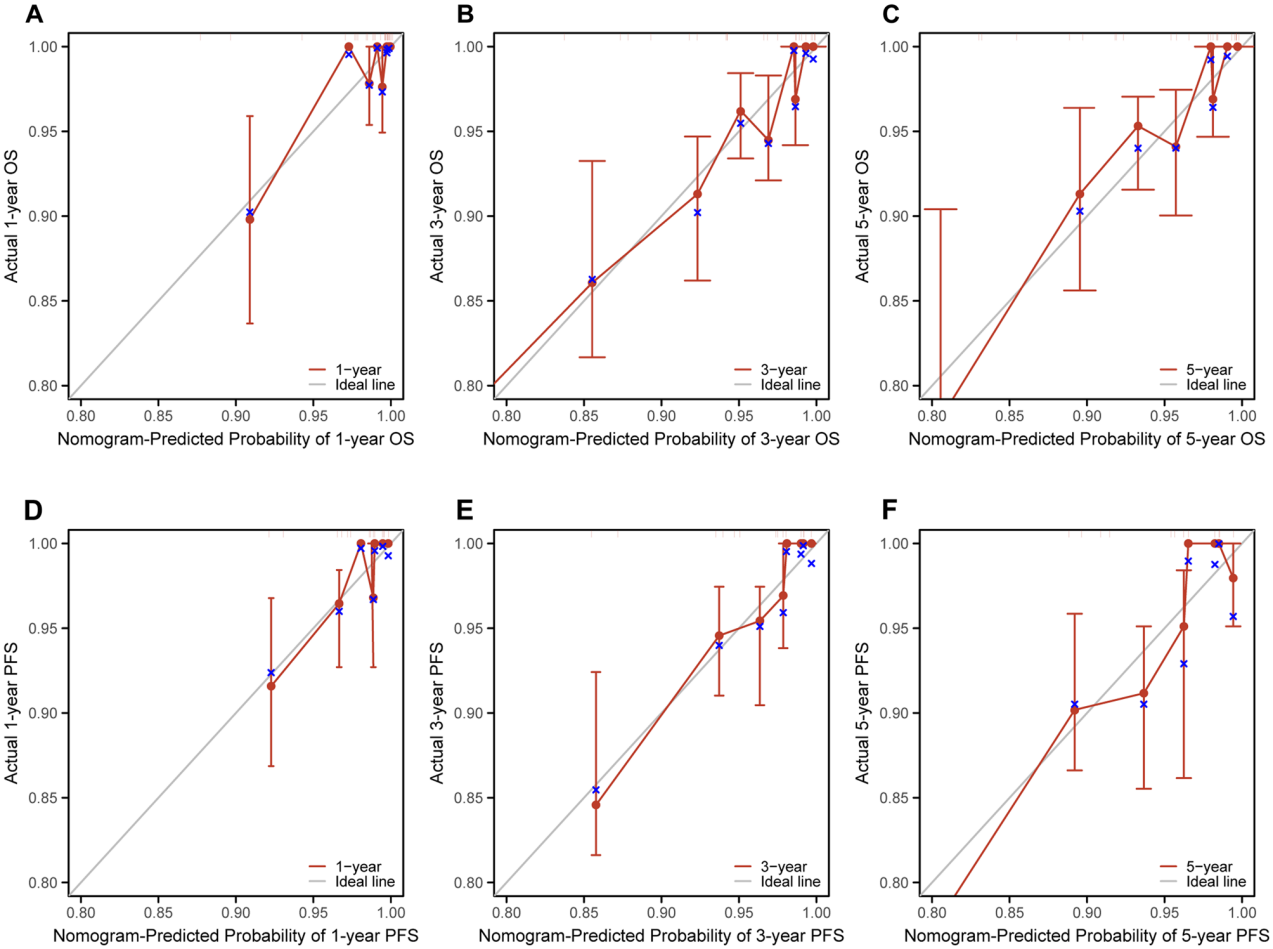
(**A**) Calibration curve for predicting 1-year OS in the training cohort. (**B**) Calibration curve for predicting 3-year OS in the training cohort. (**C**) Calibration curve for predicting 5-year OS in the training cohort. (**D**) Calibration curve for predicting 1-year PFS in the training cohort. (**E**) Calibration curve for predicting 3-year PFS in the training cohort. (**F**) Calibration curve for predicting 5-year PFS in the training cohort. The OS and PFS predicted by the nomogram models are plotted on the x-axis, and the actual OS and PFS are plotted on the y-axis.

Then, we performed internal validation of the nomograms and found that the 1-, 3-, and 5-year AUC values for ROC based on OS were 0.83, 0.84 and 0.87, respectively (Fig. 6A). The 1-, 3-, and 5-year AUC values for ROC based on PFS were 0.84, 0.86 and 0.80, respectively (Fig. 6B). The calibration curves were also applied to verify the predicted effect of the nomograms, and indicated that the calibration plots were highly consistent between the actual observation and prediction (Fig. 7).

**Figure 8:**
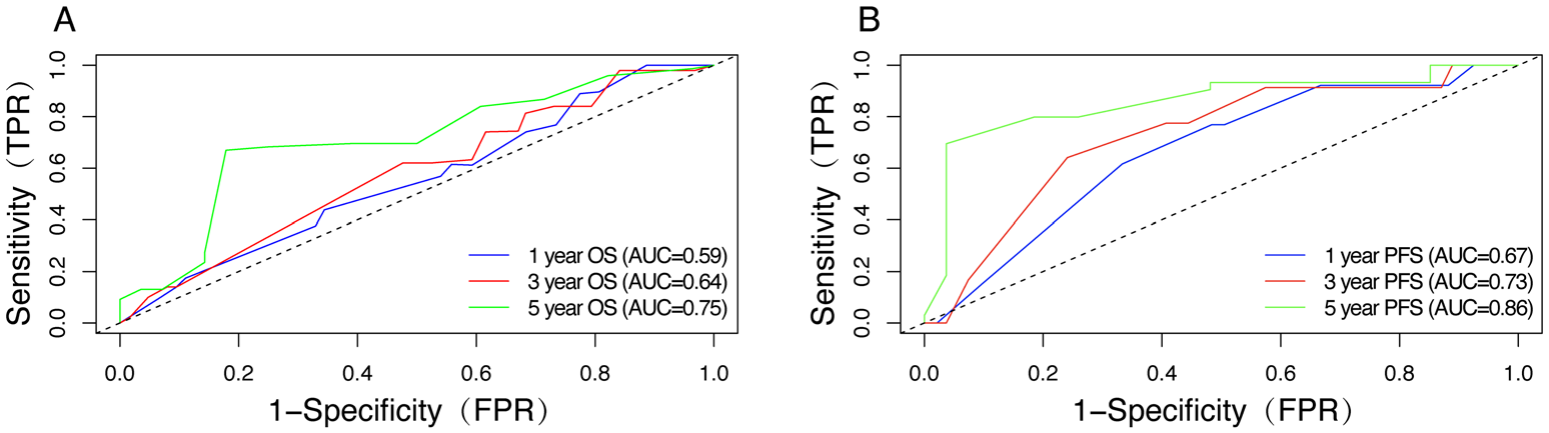
(**A**) ROC curves for 1-, 3-, and 5-year OS based on the nomogram for the validation cohort. (**B**) ROC curves for 1-, 3-, and 5-year PFS based on the nomogram for the validation cohort.

**Figure 9:**
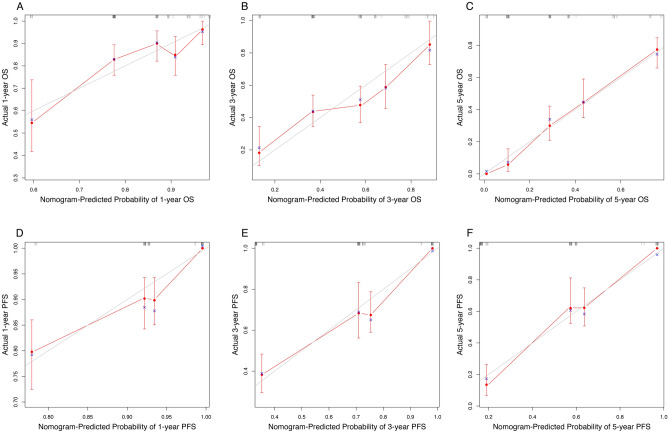
(**A**) Calibration curve for predicting 1-year OS in the validation cohort. (**B**) Calibration curve for predicting 3-year OS in the validation cohort. (**C**) Calibration curve for predicting 5-year OS in the validation cohort. (**D**) Calibration curve for predicting 1-year PFS in the validation cohort. (**E**) Calibration curve for predicting 3-year PFS in the validation cohort. (**F**) Calibration curve for predicting 5-year PFS in the validation cohort. The OS and PFS predicted by the nomogram models are plotted on the x-axis, and the actual OS and PFS are plotted on the y-axis.

The nomogram was externally validated using an independent validation cohort. The 1-, 3-, and 5-year AUC values based on OS in validation cohort were 0.59, 0.64 and 0.75, respectively (Fig. 8A). The 1-, 3-, and 5-year AUC values based on PFS in validation cohort were 0.67, 0.73 and 0.86, respectively (Fig. 8B). And the calibration plot also showed good conformity between the predicted and actual probability for OS and PFS in validation cohorts, especially for 5-year (Figs. 9). Those indicated that the constructed nomograms have good discriminatory ability for OS and PFS prediction.

## Discussion

Colorectal cancer (CRC) is the serious global public health problem. The biological behavior of tumors and patient outcomes varied widely, even at the same stage. Identifying effective prognostic factors could help develop individualized treatment strategies and improve prognosis for patients with CRC. This study evaluated the clinical significance of preoperative AGR and PNI in 396 patients with CRC. In the present study, we demonstrated that low preoperative AGR and PNI were associated with poor prognosis in CRC. AGR and PNI were both independent risk factors for OS and PFS in patients with CRC. In addition, we constructed predictive nomograms for OS and PFS.

Previous studies had shown that nutritional status in cancer patients was associated with clinical outcomes, including response to treatment and survival of patients^6,29,30^. Patients with CRC often developed malnutrition, leading to the decrease in anti-tumor immune function and cachexia, which indicated the poor prognosis^9^. Early identification of malnourished patients could improve clinical outcomes, reduce surgical complications, and prolong survival. In recent years, the immunonutritional indicators of AGR and PNI had received extensive attention. Many studies had shown that AGR and PNI were the prognostic factors for multiple types of cancers, including gastric cancer, colorectal cancer^23–25,28^.

Serum albumin (ALB) and globulin (GLB) are the two main components of serum proteins. Serum albumin is the effective indicator of patients' nutritional status and systemic inflammation^11^. Albumin has a variety of anticancer functions, including regulating cell growth and DNA replication, caching hormone homeostasis, and antioxidant effects on carcinogens such as aflatoxin^31^. In addition, albumin also plays an important role in anti-tumor therapy. Albumin can contribute to enhance tumor specificity, reduce drug induced cytotoxicity and retain concentration of the therapeutically active agent such as drug, peptide, protein, and gene for a prolonged time duration^32^. Low ALB can reflect poor nutritional status and is an independent prognostic factor for poor survival of many cancers, such as lung cancer, breast cancer, gastric cancer^11^. Globulin plays an important role in immunity and inflammation, and the high expression level of GLB is thought to be associated with tumor proliferation, immune evasion, and distant metastasis^33^. Moreover, high GLB can reflect the chronic inflammatory response and cumulative exposure to various inflammatory cytokines, which are the main characteristics of cancer cachexia^12^.

AGR combines albumin and globulin, and its expression is relatively stable, less susceptible to confounding factors such as dehydration or fluid retention. AGR may have a better predictive effect than albumin or globulin alone. AGR had been shown to be the effective prognostic factor for many types of cancers, including colorectal, gastric, breast, head and neck cancers^21–24^. Chi et al. had shown that high AGR was significantly associated with longer survival times in cancer patients^20^. AGR was the powerful independent predictor of cancer-specific long-term survival in patients with CRC^34^. Ma further found that low pretreated AGR was associated with aggressive clinicopathological features and poorer prognosis in patients with CRC^35^. This was consistent with the results of our study. In our study, low preoperative AGR was a negative prognostic factor for OS and PFS in CRC patients, and was associated with aggressive clinicopathological features such as large tumor size. After correcting for confounding factors, we found that preoperative AGR was the independent predictor of OS and PFS in CRC patients.

Malnutrition was also associated with immunosuppression^10^. ALB and GLB were involved in cancer-associated systemic inflammatory responses and could lead to immunosuppression, including the decrease in lymphocyte count and the function deficit of lymphocyte^36^. Lymphocytes were the important component of adaptive immunity and the major effector cells of anti-tumor immunity^19^. Lymphocytes exerted antitumor effects by inducing cytotoxic T cell killing and apoptosis, and their expression levels were associated with improved survival in CRC patients^37^. Iseki also found that high lymphocyte level was associated with higher OS and PFS in CRC patients^19^. Recent studies had shown that lymphocyte count during neoadjuvant therapy correlated independently with prognosis in patients with CRC and could predict response to treatment^38,39^. Immuno-inflammatory biomarkers (IIBs), including NLR, PLR and PIV, could effectively reflect the inflammation and immune status of patients, and were negative prognostic factors in CRC patients^16–18^. In this study, we also found that low AGR and low PNI were associated with higher PLR, NLR, PIV in CRC patients.

PNI was first proposed by Onodera in 1984 with the aim to evaluate postoperative complications in patients undergoing gastrointestinal surgery. PNI is calculated from serum albumin levels and peripheral blood lymphocyte counts, which can reflect the patient's immune and nutritional status. In recent years, PNI had been shown to be associated with the prognosis of a variety of cancers, including breast, lung, colorectal, and gastric cancers^25–28^. Preoperative PNI was independently related to the prognosis of CRC patients and was associated with aggressive clinicopathological features, including large tumor size, and high TNM stage^40^. This was consistent with our study. We observed that lower preoperative PNI was associated with negative prognostic factors such as large tumor size, high NLR, and high PLR. Chen had shown that PNI could serve as an independent predictor of survival and serious postoperative complications in CRC patients^41^. And in patients with locally advanced rectal cancer receiving neoadjuvant chemoradiotherapy, higher pretreatment PNI was associated with increased disease control rates and predicted long-term prognosis^42^. In this study, preoperative PNI was an independent prognostic factor for OS and PFS in CRC patients, which was consistent with previous studies. PNI has great potential to be the effective marker to guide the stratification of CRC patients. At the same time, we found that patients with low AGR and low PNI had the lowest OS and PFS, which may be related to the highest risk of malnutrition in these patients.

In this research, we confirmed the impact of a patient's immunonutritional status on prognosis. Lower preoperative AGR and PNI are associated with poorer prognosis in CRC patients. And preoperative AGR and PNI are independent prognostic factors for OS and PFS in CRC patients. Therefore, appropriate nutritional interventions may improve patient outcomes. Previous studies had also shown that appropriate nutritional interventions had a good impact on patients' nutritional status and treatment outcomes, and could reduce postoperative complications and improve survival and quality of life^43^. Pacagnella found that perioperative supplementation with arginine can reduce the incidence of complications and a significant increase in long-term survival^44^. Supplementation with glutamine appears to support the efficacy of chemoradiotherapy treatment while reducing toxicity of the tissues and improving outcomes^44^. Another similar study also confirmed that nutritional interventions during chemotherapy in patients with CRC could improve chemotherapy tolerance and reduce the loss of skeletal muscle^45^.

The present study has some limitations. First, this retrospective study was conducted at two institutions and there may be selection bias. Second, the sample size is relatively small. Therefore, a prospective study with larger sample sizes is needed to validate the results of the present. Third, the predictive nomogram was validated. Fourth, other factors related to immunonutritional status, such as C-reactive protein, were not evaluated in this study. Finally, we evaluated preoperative AGR and PNI only once, and did not observe dynamic changes. However, a single measurement may not reflect the true impact on the results.

## Conclusion

In conclusion, our study identifies that preoperative AGR and PNI are effective immunonutritional indicators to identify the prognosis of CRC patients. We developed and validated the individualized nomograms for predicting clinical outcomes in patients with CRC. The application of AGR and the PNI in evaluating preoperative immunonutritional status might help clinicians develop more effective therapy schedules. Future prospective randomized studies are needed to confirm the importance of AGR and PNI in patients with CRC.

## Data Availability

The datasets generated and analyzed during the current study are available from the corresponding author on reasonable request.

## Abbreviations

AGR: Albumin-to-globulin Ratio
ALB: Albumin
CA19-9: Carbohydrate antigen 19-9
CEA: Carcinoembryonic antigen
CRC: Colorectal cancer
GLB: Globulins
IIBs: Immuno-Inflammatory biomarkers
NLR: Neutrophil-to-lymphocyte ratio
OS: Overall survival
PNI: Prognostic nutritional index
PLR: Platelet-to-lymphocyte ratio
PIV: Pan-immune inflammation value
PFS: Progression-free survival
